# Selection of patients for nerve sparing surgery in robot‐assisted radical prostatectomy

**DOI:** 10.1002/bco2.115

**Published:** 2021-11-09

**Authors:** André N. Vis, Roderick C. N. van den Bergh, Henk G. van der Poel, Alexander Mottrie, Philip D. Stricker, Marcus Graefen, Vipul Patel, Bernardo Rocco, Birgit Lissenberg‐Witte, Pim J. van Leeuwen

**Affiliations:** ^1^ Department of Urology Amsterdam UMC, Location VUmc Amsterdam The Netherlands; ^2^ Prostate Cancer Network Netherlands; ^3^ Department of Urology Antonius Hospital Nieuwegein The Netherlands; ^4^ Department of Urology NKI/AVL Amsterdam The Netherlands; ^5^ Department of Urology Onze Lieve Vrouw Hospital (OLV) Aalst Belgium; ^6^ Department of Urology St. Vincent's Clinic Sydney NSW Australia; ^7^ Martini‐Klinik University Hospital Hamburg‐Eppendorf Hamburg Germany; ^8^ Global Robotics Institute Florida Hospital Celebration Health Orlando Florida USA; ^9^ Department of Urology University of Modena and Reggio Emilia Modena Italy; ^10^ Department of Epidemiology and Data Science Amsterdam UMC, Location VUmc Amsterdam The Netherlands

**Keywords:** erectile dysfunction, evidence synthesis, nerve‐sparing, prostate cancer, radical prostatectomy, systematic review

## Abstract

**Context:**

Robot‐assisted radical prostatectomy (RARP) has become the standard surgical procedure for localized prostate‐cancer (PCa). Nerve‐sparing surgery (NSS) during RARP has been associated with improved erectile function and continence rates after surgery. However, it remains unclear what are the most appropriate indications for NSS.

**Objective:**

The objective of this study is to systematically review the available parameters for selection of patients for NSS. The weight of different clinical variables, multiparametric magnetic‐resonance‐imaging (mpMRI) findings, and the impact of multiparametric‐nomograms in the decision‐making process on (side‐specific) NSS were assessed.

**Evidence acquisition:**

This systematic review searched relevant databases and included studies performed from January 2000 until December 2020 and recruited a total of 15 840 PCa patients. Studies were assessed that defined criteria for (side‐specific) NSS and associated them with oncological safety and/or functional outcomes. Risk of bias assessment was performed.

**Evidence synthesis:**

Nineteen articles were eligible for full‐text review. NSS is primarily recommended in men with adequate erectile function, and with low‐risk of extracapsular extension (ECE) on the side‐of NSS. Separate clinical and radiological variables have low accuracy for predicting ECE, whereas nomograms optimize the risk‐stratification and decision‐making process to perform or to refrain from NSS when oncological safety (organ‐confined disease, PSM rates) and functional outcomes (erectile function and continence rates) were assessed.

**Conclusions:**

Consensus exists that patients who are at high risk of ECE should refrain from NSS. Several multiparametric preoperative nomograms were developed to predict ECE with increased accuracy compared with single clinical, pathological, or radiological variables, but controversy exists on risk thresholds and decision rules on a conservative versus a less‐conservative surgical approach. An individual clinical judgment on the possibilities of NSS set against the risks of ECE is warranted.

**Patient summary:**

NSS is aimed at sparing the nerves responsible for erection. NSS may lead to unfavorable tumor control if the risk of capsule penetration is high. Nomograms predicting extraprostatic tumor‐growth are probably most helpful.

## 1 INTRODUCTION

Robot‐assisted radical prostatectomy (RARP) has shown excellent oncologic outcomes for men with localized prostate cancer (PCa) but carries a substantial risk of urinary incontinence and erectile dysfunction.^1^, ^2^, ^3^, ^4^

A key determinant of functional outcome is the preservation of the neurovascular bundle (NVB) at the time of surgery. The NVB is a poorly defined anatomical structure that runs along the dorsolateral side of the prostate.^5^, ^6^ It is functionally related to the autonomic nervous system and innervates the corpora cavernosa but has also been associated with the innervation of the external sphincter complex. Preserving the NVB in men undergoing RARP has been related to improved postoperative erectile function and improved urinary continence rates the first months after surgery compared with those not undergoing nerve‐sparing surgery (NSS).^1^, ^2^, ^3^, ^4^, ^5^, ^6^, ^7^, ^8^

A concern with NSS is that close surgical preparation along the prostatic capsule may inadvertently lead to a positive surgical margin (PSM) and potentially a noncurative resection. Several studies have documented the negative impact of PSM on biochemical recurrence after RARP.^1^, ^2^, ^9^ The risk of a PSM seems most present when extracapsular tumor extension (ECE) exists.^10^ Therefore, urological surgeons are reluctant to perform NSS close to the prostate when there is a concern and uncertainty about the local extent of the cancer, and this will lead to decreased postoperative functional recovery rates. Surgeons must plan NSS by balancing the competing functional and oncological outcomes. Therefore, it is optimally important to risk‐stratify patients who opt RARP for (side‐specific) NSS or otherwise for a non‐NSS approach.

We performed a systematic review of the available literature in which (contra)indications for NSS in patients undergoing RARP were associated with oncological safety and/or functional outcomes. In this, the weight of different clinical variables, multiparametric‐magnetic resonance imaging (mpMRI) parameters and of nomograms in the decision‐making process on (side‐specific) NSS were evaluated.

## 2 EVIDENCE ACQUISITION

### 2.1 Data acquisition and search strategy

A review was performed following the Preferred‐Reporting Items for Systematic‐Reviews and Meta‐Analysis (PRISMA) statement (http://www.prisma-statement.org). The review protocol was published in the PROSPERO database.^11^ Both PubMed and Embase databases were searched for English language articles published from January 2000 until December 2020. Key Search terms included indexed terms (MeSh for PubMed; EMtree for EMBASE) as well as free‐text terms. Terms expressing “prostatectomy” were used in combination with terms comprising “nerve‐sparing.”

### 2.2 Screening of abstracts and full‐text articles

**FIGURE 1:**
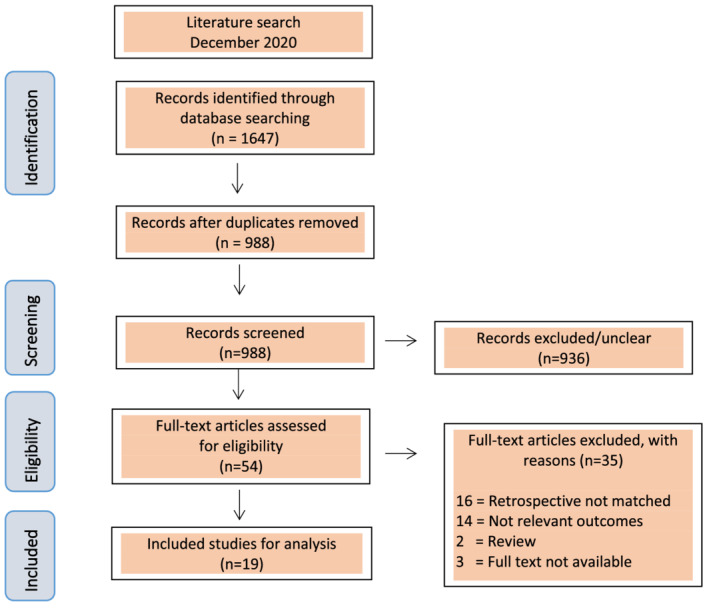
Flowchart of search strategy

We first limited our search to abstracts of studies that may be used for inclusion. Full‐text original articles were retrieved from the selected abstracts. Abstracts and original articles were independently assessed by two reviewers for eligibility (AV, PvL). Each citation was classified as inclusion, unsure, or exclusion. In case of disagreement, the manuscripts were discussed in a combined session. Agreement was obtained for all included papers. References of all full‐text articles were screened to identify additional relevant articles not found in the PubMed, EMBASE, and MEDLINE databases. Secondary publications and (systematic) reviews on a similar subject or with part of the research question as a subject were omitted, as were abstracts without accompanying full‐text articles. The final number of included and excluded studies (with the reason for exclusion) is reported in the PRISMA (Figure 1).

### 2.3 Eligibility

As proposed by the PRISMA guidelines, we used the Population, Intervention, Comparator, Outcome, and Study (PICOS) design model to direct eligibility. The specific PICO is presented in Table 1. Studies were eligible if they included patients who opted for (robot‐assisted) radical prostatectomy for histological proven PCa and mentioned specific (contra)indications for (side‐specific) NSS. As primary outcome, it was studied whether the proposed clinical and radiological variables for the (extent of) NSS were associated with oncological safety (organ‐confined disease, rates of PSM) without compromising functional outcomes (erectile function and continence rates). Studies reporting on the prediction of ECE or extraprostatic extension (EPE) using preoperative variables only without a recommendation on NSS were excluded. Second, we looked for a change of surgical plan on NSS due to application of mpMRI.

**TABLE 1:** The applied Population, Intervention, Comparator, Outcome, and Study (PICOS) design model

P	Patients	Patients opting for (robot‐assisted) radical prostatectomy
I	Intervention	(Indications for) Nerve‐sparing procedure (NSS)
C	Comparator	An unsafe nerve‐sparing procedure
O	Outcome	A safe NSS surgical procedure is defined as that performed in organ‐confined disease ± without a positive surgical margin (PSM) ± without a loss of functional outcome(s)

### 2.4 Data extraction

After full‐text evaluation, data from eligible studies were independently extracted by two reviewers. To avoid overlap of patient's populations, if multiple publications reported on the same patient population, the largest study was included. The following data were independently extracted from full‐text articles: number of patients, type of study, variables used to determine (side‐specific)NSS, and the eventual proposed conditions to perform NSS, and oncological safety and functional outcomes (Table 2).

**TABLE 2:** Outcome of studies reporting on the indications and contraindications of nerve‐sparing surgery (NSS) in men who opt for (robot‐assisted) radical prostatectomy

Reference	Number of patients	Study design	Clinical and radiological parameters, and/or nomogram	Proposed (contra) indications for NSS	Outcome(s)
Graefen (2001)	*N* = 278, prospectively validated in 353 consecutive patients	Retrospective cohort study, prospectively validated	PSA, the number of biopsies with PCa, and with dominant Gleason grade 4 and 5	Discrimination tool on (side specific) NSS based on: ‐Number of cores with Gleason grade 4/5 PCa (≤1, >1) ‐PSA (<10, ≥10) ‐Number of positive cores with PCa (≤1, >1)	The discrimination tool advises on (side specific) NSS for it correlates with organ‐confined disease in the prostatectomy specimen
Shah (2003)	*N* = 272, new algorithm prospectively validated in 263 consecutive patients	Retrospective cohort study, prospectively validated	Biopsy Gleason score, percent tumor volume, perineural invasion	No NSS if (side specific): ‐Gleason score 6 and ≥50% biopsy tumor involvement and perineural invasion ‐Gleason score 7 and ≥30% biopsy tumor involvement and perineural invasion ‐Gleason score 8 and ≥10% biopsy tumor involvement and perineural invasion	Use of this NSS algorithm decreases PSM rates, while significantly increasing the preservation of neurovascular bundles
Hricak (2004)	*N* = 135	Prospective cohort study	PSA, clinical tumor stage, biopsy Gleason score, tumor localization, percentage of positive for PCa biopsy cores, percentage of tumor involvement, MRI	Partin tables and risk of ECE were used to formulated extent of NSS from 1 (preserve) to 5 (completely resect) (not further specified)	Improved surgical planning such as on NSS with respect to organ‐confined disease due to application of MRI
Kamat (2005)	*N* = 270	Prospective cohort study	Biopsy Gleason score, length of tumor on biopsy core, location of biopsy cores with PCa	(Side specific) NSS is performed in: ‐Biopsy core with PCa <7 mm ‐Absence of core with PCa from the base of the prostate ‐Gleason score 8 or higher	Proposed criteria could assist in planning side specific NSS as EPE is often absent
Kessler (2007)	*N* = 536	Retrospective cohort study	Clinical tumor stage, positive for PCa biopsy core, number of cores with PCa per side	NSS in nonpalpable disease, no biopsy core close to the NVB, maximum one core of PCa per side	Extent of NSS is associated with improved erectile function
Zorn (2008)	*N* = 155	Retrospective cohort study	Clinical tumor stage, PSA, biopsy Gleason score, percentage of positive biopsy cores, maximal percentage of PCa on biopsy core	‐Complete NSS: cT1c, PSA ≤6, Gleason score ≤6, <33% side‐specific cores positive ‐Partial: cT2a, or cT1c with PSA >6, Gleason score 7, 33%–66% side‐specific cores positive, biopsy 33% cancer ‐None: ≥cT2b or <cT2b with Gleason score ≥8, >66% side‐specific cores positive	A side‐specific NSS protocol has reduced overall and pT2 rates of PSM. Erectile function data are not affected by nerve‐sparing protocol
Hashimoto (2010)	*N* = 82	Retrospective cohort study	PSA, clinical tumor stage, biopsy core positive for PCa in the apex, Gleason score	Algorithm on NSS using: ‐DRE (T1c, T2a, T2b vs. T2c) ‐Biopsy core in the apex (negative, positive) ‐PSA (<10 vs. ≥10) ‐Gleason score (6 vs. ≥7)	NSS caused NVB preservation without affecting PSM
McClure (2012)	*N* = 105	Retrospective cohort study	PSA, clinical tumor stage, biopsy Gleason score, number of cores with PCa, percentage of positive cores with PCa, tumor length, features on MRI	NSS was performed in those with low risk of ECE (not specified) with and without mpMRI findings	Data on MRI may improve the surgical plan to preserve or resect the NVB without compromising PSM rates
Srivastana (2013)	*N* = 1417	Retrospective cohort study	PSA, clinical tumor stage, biopsy Gleason score, MRI, intraoperative visual cues	4 risk grades of NSS based on: ‐PSA (<4, 4–10, 10–20, <20) ‐Clinical stage (T1, T2a–T2b, T2c, T3) ‐Gleason score (6, 3 + 4, 4 + 3, ≥8) and features on MRI (negative, visible, micro EPE, gross EPE) ‐Visual cues intraoperatively	Grade of NSS was associated with early return of continence
Park (2014)	*N* = 353	Retrospective cohort study	Clinical tumor stage, PSA, biopsy Gleason score, MRI	A combination of variables such as palpable tumor or not, PSA <10 and ≥10, Gleason score (<7 and ≥7), unknown assessment, with and without MRI	Data on MRI may improve the surgical plan to preserve or resect the NVB without compromising oncological outcome
Kumar (2017)	*N* = 557, high‐risk PCa	Retrospective cohort study	Clinical tumor stage, positive for PCa cores, intraoperative visual cues	‐Complete NSS: nonpalpable, <3 cores with PCa ‐Partial: non‐palpable, < 4 cores with PCa ‐None: Palpable, ≥4 cores involvement ‐Including visual cues	Selective NSS provides for reasonable intermediate term oncological and functional outcomes
Patel (2017)	*N* = 6360	Retrospective cohort study	Age, PSA, clinical tumor stage, rate of positive cores, rate of cores with Gleason >6, rate of cores with >60% of tumor positive, mean rate of tumor positive	A decision rule on NSS based on the extent of ECE using 7 clinical and biopsy variables (not specified)	Depending on the expected extent of ECE using the decision rule, the grade of (side specific) NSS was adapted
Nyarangi‐Dix (2018)	*N* = 264	Retrospective cohort study	Clinical tumor stage, PSA, ISUP grade, and MRI variables (ESUR classification for EPE, MRI volume, capsule contact length)	Nomogram for the prediction of (side specific) ECE using clinical, biopsy and radiological variables.	A predictive nomogram for ECE was developed.
Schiavina (2018)	*N* = 137	Retrospective cohort study	PSA, clinical tumor stage, biopsy Gleason score, number and location of positive PCa cores, MRI	A combination of variables (not further specified), with and without MRI findings	mpMRI improves the oncological safety of NSS and reduces PSM
Martini (2018)	*N* = 561	Retrospective cohort study	PSA, biopsy Gleason grade group, maximum percentage of tumor in the biopsy core with the highest Gleason score, ECE on mpMRI	Nomogram for the prediction of ECE using clinical, biopsy and radiological variables. The number of PSM that occur above a 10%, 15%, and 20% threshold	A predictive nomogram for ECE was developed. Using a 20% threshold, the rate of PSM is reduced
Alessi (2019)	*N* = 308	Retrospective cohort study	PSA, biopsy Gleason score, clinical tumor stage (EAU risk group), PI‐RADS on MRI, ESUR‐EPE score	Nomogram for the prediction of ECE using clinical, biopsy and radiological variables. Cut‐off levels for (side specific) NSS are not specified	The predictive nomogram could assist in (side specific) NSS in those with low risk of ECE
Jäderling (2019)	*N* = 1031	Retrospective cohort study	PSA, biopsy Gleason score, length of tumor on biopsy core, clinical tumor stage	Intrafascial, interfascial or extrafascial NSS (not specified) with and without mpMRI	Application of MRI results in more bilateral non‐NSS and results in a lower rate of PSM
Song (2020)	*N* = 314, low‐intermediate risk	Retrospective cohort study	Age, PSA, PSA‐density, free‐to‐total PSA, prostate volume, clinical tumor stage, Gleason score, PI‐RADS classification on mpMRI,	Multivariate logistic regression analysis using clinical, biopsy and radiological variables	A predictive model using PI‐RADS, PSA‐density, and biopsy Gleason score was developed and could assist in (side specific) NSS
Soeterik (2020)	*N* = 625	Retrospective cohort study	PSA, ISUP‐grade, percent highest biopsy tumor involvement, EPE on MRI,	Nomogram for the prediction of (side specific) ECE using clinical, biopsy and radiological variables. A 25% threshold of ECE is most optimal to select for (side specific), though leads to overtreatment non‐NSS in cases with organ‐confined disease	A risk assessment based on this nomogram is not recommended due to poor performance. Preservation of the NVB is associated with an increased risk of ipsilateral PSM when adjusting for patient and side specific covariates

Abbreviations: AUC, area under the curve; ECE, extracapsular extension; EPE, extraprostatic extension; ESUR, European Society of Uroradiology; ISUP, International Society of Uropathology; mpMRI, multiparametric magnetic resonance imaging; N/A, not applicable; NSS, nerve‐sparing surgery; NVB, neurovascular bundle; PI‐RADS, Prostate Imaging Reporting and Data System, in 1–5; PCa, prostate cancer; PSA, prostate‐specific antigen, in ng/ml; PSM, positive surgical margin.

### 2.5 Risk of bias

Risk‐of‐bias (RoB) assessment was performed using the Cochrane recommendations for RoB assessment of nonrandomized controlled studies (NRS).^12^ It comprises the standard Cochrane domains and additionally includes assessment of five key prespecified confounding factors for NRSs.^13^, ^14^ Potential subgroup analyses were preplanned based on the following variables: clinical tumor stage, initial prostate‐specific antigen (PSA) level, biopsy Gleason score, other biopsy variables, features‐on‐MRI, nomograms, and algorithm. Two reviewers (AV, PvL) assessed RoB. Disagreement was resolved by discussion.

## 3 EVIDENCE SYNTHESIS

### 3.1 Quantity of evidence identified

Our primary database search identified 1870 records, of which 54 full‐text articles were screened for eligibility. Finally, 19 papers met the inclusion criteria for full‐systematic review (Figure 1).^14^, ^15^, ^16^, ^17^, ^18^, ^19^, ^20^, ^21^, ^22^, ^23^, ^24^, ^25^, ^26^, ^27^, ^28^, ^29^, ^30^, ^31^, ^32^, ^33^

### 3.2 Characteristics of selected‐studies

A total of 15 840 patients were included in this review. Most eligible studies were retrospective observational cohort studies in design. Three cohort studies determined the extent of NSS on a prospective basis, based on patients' clinical and radiological variables. No randomized clinical trials (RCTs) were identified in our search process.

Nine studies assessed separate clinical, biochemical, and pathological variables to assist in recommendations on NSS,^15^, ^16^, ^17^, ^18^, ^19^, ^20^, ^21^, ^25^, ^26^ whereas six studies added MRI findings to their models^22^, ^23^, ^24^, ^28^, ^31^, ^32^ (Table 2). Four studies developed a multiparametric nomogram.^27^, ^29^, ^30^, ^33^ Seven studies associated recommendations on NSS with organ‐confinement of disease,^15^, ^16^, ^17^, ^18^, ^26^, ^27^, ^30^, ^32^ 10 with positive surgical margin or oncological outcome,^16^, ^20^, ^21^, ^22^, ^24^, ^25^, ^28^, ^29^, ^30^, ^33^ and four with functional outcomes^18^, ^19^, ^23^, ^25^ (Table 2).

### 3.3 RoB and confounding assessment of the included studies

**FIGURE 2:**
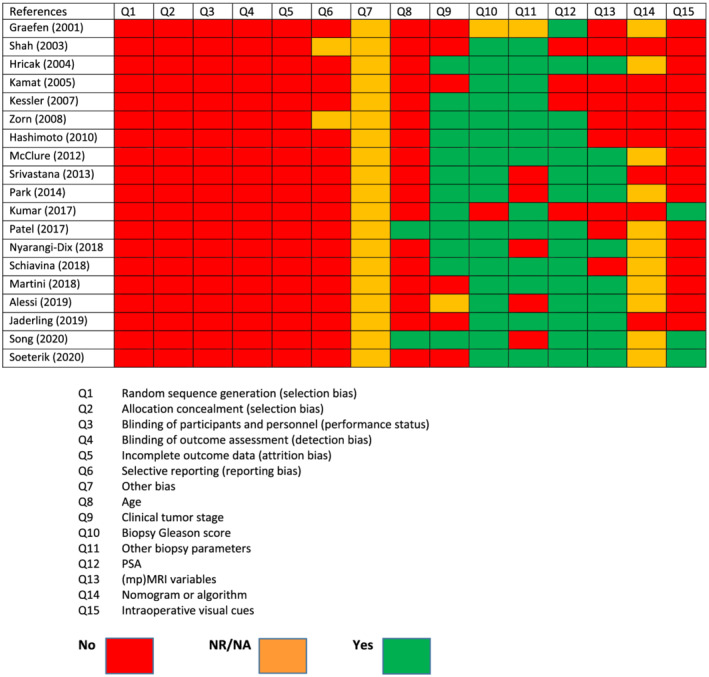
Risk of bias (RoB) assessment was performed using the Cochrane recommendations for RoB assessment of non‐randomized controlled studies (NRS). It comprises the standard Cochrane domains and additionally includes assessment of five key pre‐specified confounding factors for NRSs. NA = not available; NR = not reported

The RoB assessment of all included studies are presented in Figure 2. RoB and confounding factors were assessed for each study individually. There was high RoB for selection, performance, detection, attrition, and reporting bias, due to the lack of RCTs. Only a few studies had low to moderate confounding bias for clinical tumor stage, initial PSA, biopsy Gleason score, other biopsy variables, (mp)MRI, and nomograms/algorithms, whereas others did not report or corrected for these variables.

## 4 RESULTS OF EVIDENCE SYNTHESIS

### 4.1 Prediction models of clinical variables, mpMRI and nomograms to assist in NSS (Table 2)

Numerous papers have addressed the preoperative prediction of ECE mostly using clinical parameters and some with the addition of mpMRI, but do not specifically recommend on surgical technique or NSS.^34^ In the past, side‐specific NSS was advised only to patients with well‐differentiated PCa without a positive biopsy core at the ipsilateral side.^35^, ^36^ Thereafter, NSS was based on the number of ipsilateral positive for cancer biopsy cores and biopsy Gleason score.^16^, ^18^, ^19^, ^37^, ^38^ Graefen et al. were one of the first to develop an easy‐to‐use, objective, and reproducible discriminative tool to select patients for side‐specific NSS based on PSA and the number of cores with cancer and high‐grade cancer.^38^ Moreover, they showed for the first time that multivariate risk‐prediction based on multiple clinical variables improved the accuracy of patient selection for NSS. Since this study was published in 2001, mpMRI was not yet applied. Similarly, Zorn et al. developed a tailored‐approach on NSS based on clinical stage, biopsy Gleason score, percentage of ipsilateral cores positive for cancer, and the maximum percentage of cancer involvement.^20^ Song et al. studied 639 patients who underwent preoperative mpMRI followed by radical prostatectomy for clinically localized PCa. Based on the Prostate Imaging Reporting and Data System (PI‐RADS) of mpMRI, PSA density and biopsy Gleason score, a model was constructed that could help in the selection of suitable candidates for NSS.^32^ In line with previous works, the predictive value of these multivariate models was better than any single risk factor. Similarly, Alessi et al. made a nomogram using the European Association of Urology (EAU) classification risk group, the European Society of Uroradiology (ESUR) EPE score for any lesion in contact with the prostate capsule and the PI‐RADS score for the prediction of ECE and higher in the radical prostatectomy specimen.^30^ The nomogram was used to advise on NSS, although an area under the curve (AUC) optimum to perform NSS was not provided. Martini et al. developed a guide for nerve‐sparing RARP based on a model including 589 patients for the prediction of side‐specific ECE. Predictors in this model were PSA, highest Gleason score in the prostatic lobe, maximum percentage of cores from the highest Gleason‐grade and documented T3 on mpMRI.^29^ The performance of the multivariate model was good (AUC 82.9%) and significantly better compared with mpMRI alone (AUC 68.8%). Interestingly, they described the number of ECE cases (and corresponding PSMs) that occurred above the 10%, 15%, and 20% threshold. Using a 20% threshold, a majority of PSM could be avoided by performing non‐NSS. An external validation of the nomogram was performed with moderate‐to low discriminative capability.^39^, ^40^ Soeterik et al. further updated the nomogram of Martini and with a risk‐threshold set at 20% were able to safely perform side‐specific NSS in the majority of patients.^33^ Nyarangi‐Dix et al. developed a nomogram in 264 surgically treated patients which was largely based on Gleason grade, preoperative MRI‐findings including prostate volume, ESUR‐classification for EPE, and tumor capsular contact length.^27^ Despite performing well, it was not validated externally. From a Japanese study group, a simple decision‐making algorithm on NSS was developed using clinical tumor stage (T1c‐T2b vs. T2c), PSA (≥10 ng/ml vs. <10 ng/ml) and apical biopsy Gleason score (≥7 vs. <7). Despite easy‐to‐use, the algorithm was not prospectively evaluated.^21^

Srivastana et al. from the group of Ash Tewari proposed a risk‐stratified grade of (side‐specific) NSS in which PSA, clinical tumor stage, biopsy Gleason score, and findings on mpMRI determined the extent of NSS along with visual cues intraoperatively.^23^ Although a large number of patients was studied, this study was retrospective in design, with the grade of NSS being a subjective measure and not being standardized.

### 4.2 Change of surgical plan on NSS due to application of mpMRI

One of the earlier studies evaluating MRI on surgical plan evaluated 144 patients undergoing radical prostatectomy.^17^ This study group showed that when a preoperative probability of <25% of organ‐confined disease was assessed using the Partin‐staging tables, MRI changed the surgical plan in 78% of patients and favored NSS in 83% of patients. MRI had shown to have an incremental value in the clinical assessment additive to currently applied nomograms, although in this series functional‐imaging by dynamic contract enhanced (DCE) imaging and diffusion weighted imaging (DWI) was not applied. Using the same criteria as Martini et al. for the extent of NSS, Schiavina et al. studied two cohorts of patients and determined whether mpMRI changed the surgical plan on NSS. Indeed, mpMRI changed surgical plans equally between the direction of more radical and a less radical approach.^28^ Unfortunately, it was not clear based on what features the extent of surgery was chosen. Jägerling et al. found in their series of 1037 surgically treated patients that mpMRI was associated with an increased chance of undergoing bilateral non‐NSS versus any type of NSS (RR1.84 [95% CI 1.11–3.03]). There was a slightly increased chance of undergoing bilateral NSS in those who did not undergo mpMRI (RR1.09 [95% CI 1.00–1.20]) compared with unilateral or non‐NSS.^31^

Park et al. studied 353 men who underwent radical prostatectomy with a preoperative mpMRI where the surgeon determined preoperatively the degree of NSS based on PSA level, that is, NSS in those with PSA < 10 ng/ml, nonpalpable tumors and a biopsy Gleason score below 7. They determined NSS without incorporating mpMRI findings and then once again after reviewing the mpMRI. The surgical plan was changed in 26% of the patients, to either a more aggressive NSS approach (57%) or a wider margin of resection (43%). In patients with intermediate and high‐risk features, a change of surgical plan was made more often after reviewing the mpMRI, that is, in 31% and 40% of cases, respectively.^24^ Similarly, McClure et al. showed that the initial surgical plan was changed in 28 of 104 (27%) patients after reviewing the MRI.^22^ The surgical plan was changed to NSS in 17 out of 28 patients (61%) and to a non‐NSS in 11 (39%). In patients whose surgical plan was changed to NSS, there were no PSM on the side of the prostate with a change in treatment plan.

## 5 DISCUSSION

RARP is the main curative surgical approach in men with localized PCa. Despite providing a high chance of cancer control, RARP is associated with a nonnegligible risk of erectile dysfunction and urinary incontinence.^1^, ^2^ These downsides of RARP may have a detrimental impact on a patients' health‐related quality of life (HRQoL) during follow‐up.^41^, ^42^, ^43^ Advances in surgical technique and understanding of the pelvic floor anatomy have allowed for the preservation of the NVB, which have repeatedly been shown to improve functional outcomes after surgery.^1^, ^2^, ^3^, ^4^, ^5^, ^6^, ^7^ NSS is particularly aimed to preserve these bundles and to prevent the negative sequelae of RARP and maintain HRQoL after surgery. We performed a systematic review to investigate the indications for NSS in RARP and to study the use of various clinical variables and mpMRI and of nomograms in the decision‐making process on (side‐specific) NSS.

Most currently applied PCa guidelines recommend NSS primarily in younger men with adequate erectile function.^44^, ^45^, ^46^, ^47^, ^48^ Different meta‐analyses demonstrated that patients undergoing NSS had improved continence rates in the first months after RARP, but the studies come to different conclusions for the period after 6 months.^1^, ^2^, ^3^, ^4^, ^5^ It has been argued, however, that even early improvement in urinary continence (<3 months after surgery) is enough to perform NSS with respect to the improvement in HRQoL.^49^ Therefore, NSS might be performed regardless of potency status.^19^ Despite these recommendations, few studies have addressed age and preoperative functions into their predictive models.

In the PCa guidelines of the EAU and American Urological Association (AUA), it is advocated that NSS should be offered to patients with localized PCa undergoing RARP.^44^, ^45^ The concept of locally confined versus locally advanced disease, however, is poorly defined. Commonsensically, one should refrain from NSS in the presence of a tumor that extends through the prostate capsule and grows into the NVB. Sparing the NVB would inevitably lead to a PSM and thereby to biochemical and/or local recurrence of disease.^10^, ^50^ As such, the selection for NSS comes down to accurate local tumor staging and the ability to define the precise tumor's anatomy.

Historically, tumor staging relied heavily on digital rectal examination (DRE), transrectal ultrasound (TRUS) imaging, and the biopsy Gleason score. Unfortunately, preoperative clinical staging based on DRE and biopsy as separate variables has limited accuracy with understaging of locally extensive disease in a substantial number of cases.^51^, ^52^ Clinical variables were often combined into algorithms such as the Partin tables and the Ohori, Steuber, Briganti, and Memorial Sloan Kettering Cancer Center (MSKCC) nomograms.^53^, ^54^, ^55^, ^56^, ^57^, ^58^ The advent of novel imaging tools, particularly mpMRI, was believed to improve clinical staging and to assist in proper surgical planning such as resection of the NVB or NSS.^28^, ^59^, ^60^ However, in a recent meta‐analysis involving 9796 patients from 75 different studies, the sensitivity of mpMRI for ECE reached 0.57 only (95% CI 0.49–0.64), with a specificity of 0.91 (95% CI 0.88–0.93).^61^ This implies that ECE is present in a substantial proportion of patients who showed no signs of capsule penetration on mpMRI. Consequently, the sensitivity of mpMRI as a single variable seems inadequate to safely exclude the presence of T3 tumors and to perform NSS accordingly.^62^

The use of nomograms is recommended by different PCa guidelines to improve preoperative risk assessment of ECE.^44^, ^45^, ^46^ Nomograms allow to make complex risk estimations, outperforming individual clinical parameters. The above‐mentioned probability nomograms considered PSA, clinical tumor stage, and biopsy Gleason score, but not mpMRI. Adding mpMRI to these models has further improved the predictive accuracy for ECE.^63^ Indeed, several retrospective studies used either serum PSA, biopsy Gleason score, other prostate biopsy variables, and PI‐RADS score on mpMRI to predict organ‐confined disease. So, the predictive capability of multivariate prediction models instead of single parameters such as (side‐specific) biopsy Gleason score or mpMRI has the best predictive performance for non‐organ‐confined disease and may guide surgeons into proper decision making on NSS. However, from most of these studies, we were unable to find clear risk thresholds that may help clinicians in their decision to perform or to refrain from NSS. In a theoretical proposal, Lepor et al. were one of the few who made recommendations on specific thresholds to perform or to refrain from NSS. By applying a calculation using the location of ECE and the risk of PSM in ECE, cases with a ≥30% risk of ECE should be withheld NSS.^64^ A (side‐specific) threshold of 20% under which NSS could be safely performed was proposed using nomograms incorporating both clinical parameters and readily applicable mpMRI variables.^33^, ^40^ This threshold may be altered based on patients (oncological) objectives, age, and baseline erectile function.

Recent recommendations suggest that the extent of ECE may determine the approach to and the extent of NSS. Dissections performed closer to the prostate (e.g., extrafascial, interfascial, and intrafascial) and bilaterally instead of unilaterally associated with superior functional outcomes.^25^, ^65^, ^66^, ^67^ Patel et al. developed a decision rule to assist surgeons in their decision‐making process on NSS based on the extent of ECE. Based on 6360 surgically treated patients, a predictive algorithm of the (side‐specific) width of ECE (in mm) was constructed providing a suggestion to the surgeon regarding the boundaries of the resection.^26^ Unfortunately, a clear association of the graded approach to NSS with functional outcomes was not reported. NSS in patients with high‐risk features (i.e., Gleason grade ≥4 or >cT2) was often discouraged as the risk of ECE and PSM is increased. Kumar et al., however, indicated in a retrospective study that selective partial or complete NSS was still feasible in a series of high‐risk patients with PCa with oncological outcomes that proved similar to those who underwent non‐NSS.^26^

It is important to state that in a systematic review and meta‐analysis including 124 studies with 73 448 patients, NSS did not increase PSM rates, nor did it compromise cancer control if patients were carefully selected depending on tumor location, size, and grade.^4^, ^68^ However, in a large multicenter study including 2574 patients, Soeterik et al. showed that NSS was associated with an increased risk of PSM when side‐specific PSM was concerned.^33^ So, in patients with a specific wish to maintain erections, adequate counseling on the possibility of partial and unilateral/bilateral NSS and on functional recovery and oncological outcome after NSS is mandatory.

Despite the finding that existing nomograms may help urological surgeons to plan on NSS, a significant proportion of NSS techniques during RARP are changed intraoperatively. Reasons for deviation in technique can include challenging anatomical features (e.g., narrow pelvis and fatty tissues), disrupted surgical planes due to previous treatments or septicemia, and other factors leading to more difficult resections.^69^ Couture et al. showed that 46.9% of surgically treated cases had a change of surgical plan intraoperatively, in which increasing age was shown to be the most significant variable responsible for a change of management. The authors stated that surgeons are less likely to perform NSS in older patients in whom surgical planes are difficult to locate. Other variables associated with a higher rate of conversion of surgical plan were postbiopsy septicemia, high unilateral biopsy Gleason score, and larger prostates. Interestingly, the number of lifetime biopsies was not a significant predictor of unplanned non‐NSS, a particularly novel and valuable finding for patients under active surveillance.^69^

Furthermore, only few of the decision‐making tools for EPE have been externally validated within 5 years after development and often perform poor.^34^ Adding mpMRI‐findings may potentially improve the predictive performance of these nomograms.^70^ Though, urological surgeons need to consider the limitations of these decision tools when applying them on their own patients.^34^

The efficacy and safety of perioperative Neurovascular Structure Adjacent Frozen Section Examination (NeuroSAFE) is being investigated in RCTs.^71^, ^72^, ^73^ Nonrandomized studies have shown that NeuroSAFE is able to improve NSS rates while it may help to achieve a modest reduction in PSM rates. Functional outcomes and long‐term oncological outcomes need to be further explored. However, the establishment of the respective infrastructure to routinely perform the NeuroSAFE investigation intraoperatively is labor intensive and not possible in every institution. Furthermore, the feasibility of 3D imaging techniques and augmented‐reality using preoperative mpMRI incorporated into the robotic systems and/or the use of 3D reconstructions and 3D prints of the prostate is investigated and could possibly assist the surgeon into making a proper surgical plan on the side and extent of NSS.^74^, ^75^ At last, implementing modern PSMA‐PET imaging into predictive nomograms, according to the newly developed e‐PSMA guidelines, may be used to predict the side of ECE, increase the rate of (side‐specific) NSS, lower PSM rates, and improve functional outcomes even in non‐organ‐confined disease.^76^

This review was performed robustly in accordance with recognized standards, with a broad search strategy designed by a statistician/bio‐epidemiologist. Limitations include the retrospective, single‐institution nature of the majority of included studies. The absence of RCTs and the overall significant clinical and methodological risk of bias across studies make the quality of the evidence inherently low.

## 6 CONCLUSIONS

Current literature suggests that NSS should not be performed in patients with a high likelihood of (extensive) ECE. At present, the use of preoperative multiparametric nomograms including mpMRI is suggested, and these nomograms are applied with improved diagnostic accuracy than single clinical, pathological, or radiological variables. Controversy exists on risk thresholds and on decision rules for a conservative versus a less conservative surgical approach of NSS. Individual clinical judgment may still consider the patient's specific wish to maintain erections and the patient's consent for potentially discounting cure.

## AUTHOR CONTRIBUTION

**A.N. Vis:** Design, writing and statistical analysis. **R.C. van den Berg:** Design, writing and supervision. **H. van der Poel:** Writing and review. **A. Mottrie:** Writing and review. **V. Patel:** Writing and review. **P. Stricker:** Writing and review. **M. Graefen:** Writing and review. **B. Rocco:** Writing and review. **B. Lissenberg‐Witte:** Statistical review. **P. van Leeuwen:** Design, writing, statistical analysis, supervision.
